# Using optically pumped magnetometers to measure magnetoencephalographic signals in the human cerebellum

**DOI:** 10.1113/JP277899

**Published:** 2019-07-18

**Authors:** Chin‐Hsuan Lin, Tim M. Tierney, Niall Holmes, Elena Boto, James Leggett, Sven Bestmann, Richard Bowtell, Matthew J. Brookes, Gareth R. Barnes, R. Chris Miall

**Affiliations:** ^1^ Wellcome Centre for Human Neuroimaging Queen Square Institute of Neurology University College London London UK; ^2^ School of Psychology University of Birmingham Birmingham UK; ^3^ Sir Peter Mansfield Imaging Centre School of Physics and Astronomy University of Nottingham Nottingham UK; ^4^ Department of Clinical and Movement Neuroscience Queen Square Institute of Neurology University College London London UK

**Keywords:** optically pumped magnetometer, magnetoencephalography, cerebellum, eyeblink conditioning

## Abstract

**Key points:**

- The application of conventional cryogenic magnetoencephalography (MEG) to the study of cerebellar functions is highly limited because typical cryogenic sensor arrays are far away from the cerebellum and naturalistic movement is not allowed in the recording.
- A new generation of MEG using optically pumped magnetometers (OPMs) that can be worn on the head during movement has opened up an opportunity to image the cerebellar electrophysiological activity non‐invasively.
- We use OPMs to record human cerebellar MEG signals elicited by air‐puff stimulation to the eye.
- We demonstrate robust responses in the cerebellum.
- OPMs pave the way for studying the neurophysiology of the human cerebellum.

**Abstract:**

We test the feasibility of an optically pumped magnetometer‐based magnetoencephalographic (OP‐MEG) system for the measurement of human cerebellar activity. This is to our knowledge the first study investigating the human cerebellar electrophysiology using optically pumped magnetometers. As a proof of principle, we use an air‐puff stimulus to the eyeball in order to elicit cerebellar activity that is well characterized in non‐human models. In three subjects, we observe an evoked component at approx. 50 ms post‐stimulus, followed by a second component at approx. 85–115 ms post‐stimulus. Source inversion localizes both components in the cerebellum, while control experiments exclude potential sources elsewhere. We also assess the induced oscillations, with time‐frequency decompositions, and identify additional sources in the occipital lobe, a region expected to be active in our paradigm, and in the neck muscles. Neither of these contributes to the stimulus‐evoked responses at 50–115 ms. We conclude that OP‐MEG technology offers a promising way to advance the understanding of the information processing mechanisms in the human cerebellum.

## Key points

- The application of conventional cryogenic magnetoencephalography (MEG) to the study of cerebellar functions is highly limited because typical cryogenic sensor arrays are far away from the cerebellum and naturalistic movement is not allowed in the recording.
- A new generation of MEG using optically pumped magnetometers (OPMs) that can be worn on the head during movement has opened up an opportunity to image the cerebellar electrophysiological activity non‐invasively.
- We use OPMs to record human cerebellar MEG signals elicited by air‐puff stimulation to the eye.
- We demonstrate robust responses in the cerebellum.
- OPMs pave the way for studying the neurophysiology of the human cerebellum.

## Introduction

Our understanding of cerebellar function has undergone a paradigm shift in recent decades due to studies of neuroanatomy (Glickstein *et al*. 2011) and neuropsychology (Schmahmann & Sherman, 1998) and functional magnetic resonance imaging (fMRI) (Stoodley & Schmahmann, 2009; Buckner, 2013). Until recently thought of as part of only the motor system, the cerebellum is essential for a variety of cognitive and social functions as indicated by accumulating evidence (Sokolov *et al*. 2017). Despite this expanding repertoire of cerebellar functions, there is a marked absence of human electrophysiological studies in this area.

In the domain of magnetoencephalography (MEG), a small body of research has documented cerebellar evoked potentials (Tesche & Karhu, 1997, 2000; Martin *et al*. 2006; Houck *et al*. 2007) or activity as a part of physiological (Gross *et al*. 2002; Tass *et al*. 2003; Pollok *et al*. 2004, 2005; Muthukumaraswamy *et al*. 2006; Jerbi *et al*. 2007) or pathological (Timmermann *et al*. 2003; Schnitzler *et al*. 2009) oscillatory networks. These MEG studies provided important insights. Nonetheless, they are scarce in comparison with other types of studies, especially fMRI, and have used limited varieties of tasks. It should also be noted that many of them reported cerebellar activation, but without examining or discussing it in detail. This poverty of reports is due to several factors. First, compared to the cerebral cortex, the cerebellar cortex is less favourable to the generation of a measurable MEG signal. Its densely folded anatomy causes a high degree of field attenuation due to locally opposing current sources (Tesche & Karhu, 1997; Hashimoto *et al*. 2003; Dalal *et al*. 2013). Second, cerebellar neurons are thought to have low firing synchrony, based on the small amplitudes observed in local field potential studies (Gerloff *et al*. 1996). Third, the majority of the cerebellum is located deep in the human cranium, so any electromagnetic sources are distant from typical SQUID‐based MEG sensors. These factors combined make it not unexpected that MEG signals generated in the cerebellum are smaller and less likely to be distinguishable compared to those produced by the neocortex. Further, to maintain signal quality and minimize co‐registration errors, cryogenic MEG requires subjects to remain very still and thus limits the application of many naturalistic movement paradigms relevant to cerebellar dysfunction (e.g. the finger–nose–finger test). The issue is not exclusive to MEG – to date, few studies with scalp cerebellar EEG have been reported (Muthukumaraswamy *et al*. 2003; Lascano *et al*. 2013; Todd *et al*. 2018). It is widely assumed that scalp EEG recordings for the cerebellum suffer from muscle artefacts (Muthukumaraswamy, 2013). Intracranial recordings in the cerebellum are also rare because of their limited clinical indications (Niedermeyer, 2004; Dalal *et al*. 2013). As a result, the electrophysiology of the human cerebellum is largely understudied compared to the neocortex.

The recent development of optically pumped magnetometers (OPMs) as a tool for MEG provides a new opportunity to investigate cerebellar electrophysiology. OPMs are high sensitivity magnetic field sensors. They do not need cryogenic cooling so can be flexibly placed on the scalp. We have recently built such a wearable OP‐MEG system, placing the sensors close to the scalp by mounting them in a 3D printed cast based on the individual's head MR imaging (Boto *et al*. 2017, 2018). The use of a 3D‐printed cast can accurately inform sensor positions and orientations with respect to the brain anatomy, thus facilitating accurate source imaging. This system potentially also reduces the sensor‐to‐brain distance to enhance signal magnitude (Boto *et al*. 2017). These sensors can be positioned in a dense array over a specific brain region of interest (Tierney *et al*. 2018), although at present only in modest numbers. Importantly, in combination with a field‐nulling apparatus (Holmes *et al*. 2018; Iivanainen *et al*. 2019), one can minimize magnetic field variation at the sensors due to head movement in the ambient field. Besides, this system is less susceptible to muscle artefacts compared to EEG (Boto *et al*. 2018). These characteristics combined allow non‐invasive mapping of human electrophysiology when subjects interact with the real world and even move freely (Boto *et al*. 2018), thus making OP‐MEG an ideal candidate for the study of cerebellar electrophysiology of both motor and non‐motor tasks.

As a proof‐of‐concept study, we recorded OP‐MEG data while delivering non‐noxious air‐puffs to the eye to trigger blinks. Air‐puffs are the unconditioned stimuli (US) in a well‐established cerebellar associative learning paradigm: eyeblink conditioning. In single‐unit recording experiments in animal models, the stimuli elicit activity in the principal cells of the cerebellum, the Purkinje cells. In untrained animals (Ohmae & Medina, 2015) these cells show both ‘simple spike’ responses driven by mossy fibre inputs from the brainstem pontine nuclei, and ‘complex spike’ responses to climbing fibres that project from the inferior olive to bilateral, predominantly ipsilateral, cerebellar cortices. Animals and humans present comparable behavioural responses to physiological and pathological (cerebellar lesion) interventions in this paradigm (see Freeman & Steinmetz (2011) for a review of the neural circuitry based on animal studies; see Gerwig *et al*. (2007) for a review of human lesion studies). Functional MRI studies in humans also accord well with corresponding electrophysiological studies in animals, with a prominent blood oxygenation level‐dependent response predominantly in the ipsilateral cerebellar cortex (Dimitrova *et al*. 2002; Cheng *et al*. 2008; Thurling *et al*. 2015). Consequently, cerebellar activation may well be expected in our OP‐MEG recording.

We aim here to demonstrate that neural signals in the cerebellum, which have been observed in response to air‐puff stimulation in both the invasive animal and non‐invasive human literature (e.g. fMRI), can be measured with OP‐MEG. We present evoked responses localized to the cerebellum, and other physiological signals relevant to the stimulation, including eye‐blinks, evoked responses in the somatosensory cortex and induced responses localized to the occipital area (Bardouille *et al*. 2006; Liu *et al*. 2017). Taken together, we show that wearable OP‐MEG provides a promising future to examine the cerebellum during human cognition and action, and in pathological conditions linked to cerebellar dysfunction.

## Methods

This section is divided into three parts. First, we describe the OP‐MEG system. Second, we summarize the experimental procedures for cerebellar activity measurement. Finally, we introduce the inversion scheme used to localize the source activity.

### Ethical approval

The study conformed to the standards set by the *Declaration of Helsinki*, except for registration in a database. The protocol was approved by the University of Nottingham Medical School Research Ethics Committee and the University of Birmingham Research Ethics Committee. Written informed consent was obtained from all participants. The experiments took place at the University of Nottingham.

### Participants

Three healthy subjects (1 female, 2 male) aged 27–50, with no history of psychiatric or neurological diseases, participated in the study. All subjects were naïve to the eyeblink conditioning.

### OP‐MEG system

**Figure 1:**
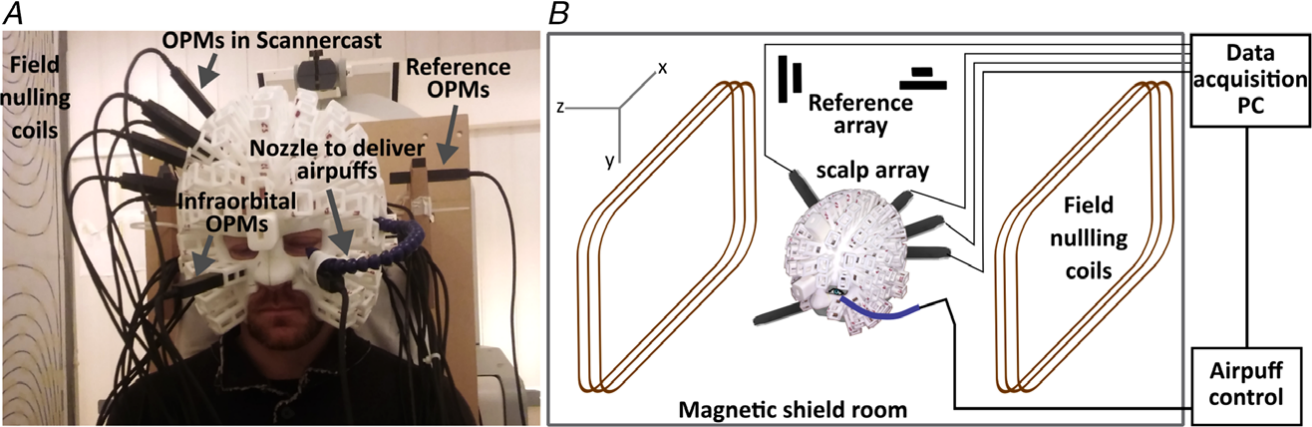
Experimental set‐up (*A*) and schematic illustration (*B*) of the eyeblink paradigm for cerebellar activity measurement The participant is seated inside a magnetically shielded room (MSR) wearing a customized scanner‐cast. Air‐puffs are delivered to the outer canthus of the participant's left eye via a nozzle. OPM sensors are inserted into slots covering the cerebellum and right somatosensory cortex. Two more sensors are placed in bilateral infra‐orbital slots to measure eyeblinks. Field‐nulling coils standing either side of the participant carry currents set so as to minimize the residual magnetic field in the MSR, which is measured by 4 reference OPMs prior to scanning. The task‐controlling laptop, located outside the MSR, sends synchronizing triggers that are recorded by the data acquisition PC during scanning. [Color figure can be viewed at wileyonlinelibrary.com]

The OP‐MEG system has been previously described in detail (Boto *et al*. 2017, 2018; Holmes *et al*. 2018; Tierney *et al*. 2018). Briefly, the system consists of an OPM sensor array within a customized cast of the head, and a field‐nulling apparatus comprising four reference OPM sensors and field‐nulling coils (Fig. 1).

#### Optically pumped magnetometer

The OPM sensors used here (QuSpin Inc., Louisville, CO, USA) have previously been described in detail (Shah & Wakai, 2013; Osborne *et al*. 2018). In brief, each OPM sensor incorporates three key components: a 795 nm wavelength laser, a 3 × 3 × 3 mm^3^ cell containing rubidium‐87 (^87^Rb) vapour, and a photodiode. The laser spin‐polarizes the ^87^Rb atoms in the direction of the laser beam. The photodiode perceives the intensity of the laser transmitting through the cell. At zero magnetic field, the cell is transparent to the laser light and the photodiode detects a maximal signal. A small local field (due to brain activity) causes Larmor procession of the ^87^Rb atoms, which decreases the transparency of the cell because some laser light is absorbed by the atoms. The output of the photodiode shows a zero‐field resonance with Lorentzian line shape, allowing the measurement of the field (Dupont‐Roc *et al*. 1969). In practice, the sensors have a bandwidth of approximately 0–130 Hz with a noise level of ∼15 fT/√Hz in the 1–100 Hz band, and a dynamic range of ±1.5 nT.

Each OPM sensor also contains a set of three coils which generate three orthogonal fields to cancel out the static field inside the vapour cell up to ∼50 nT. These on‐sensor coils compensate the ambient environmental field in a typical magnetic shield room (MSR) and allow sensors to operate at a fixed position and orientation. To keep the OPMs within their dynamic range during naturalistic movement, we further apply an array of bi‐planar coils to null the residual field inside the MSR (see the field nulling apparatus section).

#### Field nulling apparatus

The earth's residual static field in the Nottingham MSR is ∼25 nT with a maximal gradient of approximately 10 nT/m. This means that even minimal head movements (e.g. a 4‐degree rotation) can easily generate signals which exceed the OPM's dynamic range. In order to mitigate these effects, we used a set of bi‐planar coils to generate magnetic fields which counteract the remaining static field as measured by four reference sensors close to the head. By applying this method, the dominant components of the static field and field gradient can be diminished by factors of 46 and 13, respectively, in a volume of 40 × 40 × 40 cm^3^ encapsulating the head (Boto *et al*. 2018; Holmes *et al*. 2018).

#### Helmet design

Data from an anatomical T1‐weighted MRI scan were used to generate a 3D mesh representing the outer surface of each participant's scalp. This 3D mesh was used in 3D printing to shape the inner surface of a nylon ‘scanner‐cast’, as described (Boto *et al*. 2017; Meyer *et al*. 2017; Tierney *et al*. 2018), with sockets around the outer surface to hold the OPM sensors. Importantly, the mesh and subsequently produced wearable helmet provide accurate socket and thus sensor positions and orientations, which are in the same coordinate space as the MRI image. Therefore, no coregistration is needed and the sensor positions are defined as the centre of the socket bases with a 6.5 mm offset, which takes into account the distance between the outside of the sensor housing and the centre of the vapour cell (Osborne *et al*. 2018). Here, with limited numbers of sensors available, we placed the sensors proximal to the cerebellar and somatosensory cortices. Figure 1 shows the sensor configuration in a typical subject. Across the three participants, 13–19 posterior sensors and 4–6 somatosensory sensors were used. Additionally, two sensors were placed in bilateral infra‐orbital slots for eye‐blink detection (Fig. 1 *A*).

### Experiment: eyeblink paradigm

Eyeblinks were elicited by a 32 ms air‐puff delivered through a nozzle mounted on the scanner‐cast (Fig. 1 *A*), essentially a pressurized air cylinder (1 bar) fed into a 10 m semi‐rigid plastic tube (2 mm internal diameter), under the control of a bespoke pneumatic valve controller. Some details of the pneumatic delivery system are given in Leonardelli *et al*. (2015). The nozzle directed the air‐puff to the outer canthus of the left eye from a distance of approximately 2–4 cm, individually adjusted to evoke a visible blink after each delivery, but without discomfort. The arrival time of the air‐puff was calibrated off‐line using a microphone and was relatively insensitive to the distance of the nozzle over a limited range (∼1 ms/cm). Subjects received four contiguous 12 min blocks of stimulation. To equate the task to the baseline phase of a previously validated eyeblink conditioning paradigm (Cheng *et al*. 2008), each block constituted 200 trials: 140 trials of air‐puffs, 50 trials of a 550 ms binaural tone (2800 Hz) and 10 paired trials with the tone co‐terminated with air‐puff delivery; trial order was randomized in sets of 20. A total of 600 air‐puff trials were recorded per subject. Every trial began with a random wait of 1–2.5 s to avoid habituation and anticipation; inter‐stimulus intervals averaged to 3.6 s.

To understand the relationship between neck muscle and OP‐MEG activity, two additional blocks, with surface electromyography (EMG) recorded by MEG‐compatible electrodes available on the CTF275 MEG in the Nottingham MSR, were administered in one subject (subject 3) on a separate day. EMG electrode pairs were placed over the bilateral cervical splenius capitis (SPL), the primary muscles activated during neck extension, based on Sommerich *et al*. (2000). Eleven posterior OPM sensors were used in this recording.

### OP‐MEG data collection

The OP‐MEG data were digitized at a sampling rate of 1200 Hz, using a 16‐bit digital acquisition (DAQ) system (National Instruments, Austin, TX, USA) controlled by custom‐written software in LabVIEW (National Instruments). The air‐puff trigger signals were also acquired through the same DAQ system and sent to both the OPM and CTF MEG (during sessions with concurrent EMG) data acquisition PCs to synchronize air‐puff triggers, OP‐MEG and EMG data.

### Data analysis

All of the data analysis was performed using SPM12 within the MATLAB environment (Release 2014b, The Mathworks Inc., Natick, MA, USA).

#### OP‐MEG data pre‐processing

OP‐MEG data were filtered between 5 and 80 Hz (or 0–200 Hz for eyeblink analysis, explained in more detail below) and each trial epoched between −1000 and +1000 ms relative to air‐puff onset. Because OPMs are configured as magnetometers (as distinct from gradiometers which are used in many cryogenic MEG systems), they are susceptible to increased environmental interference. We mitigated interference by constructing virtual gradiometers, which linearly regress the signal recorded by the reference array from the signal recorded at the scalp array (Fife *et al*. 1999; Boto *et al*. 2017). Thereafter, data were concatenated across blocks and all trials of data were ranked according to signal variance. Trials with variances higher than [median + 3 × median absolute deviation] were rejected (Leys *et al*. 2013). For the evoked response analysis, the remaining trials (548, 588 and 585 trials with air‐puff stimulation, for subject 1, 2 and 3 respectively) were baseline corrected to the mean of the window 50 ms prior to stimulus onset and then averaged. Subject‐specific early and late peaks were identified through global field power amplitudes. Paired Student's *t* test was used to analyse the changes of amplitudes between baseline and evoked responses; *P* < 0.05 was considered significant.

#### Spectral analysis

For induced spectral power changes, single trial time–frequency (TF) decompositions in sensor space were calculated for each subject using a Morlet wavelet transform (Tallon‐Baudry *et al*. 1998) and then averaged across trials and the power of the evoked responses subtracted out. The wavelet transform was calculated for each time point between −1000 and +1000 ms, with 76 scale bins corresponding to frequencies between 5 and 80 Hz. For each trial and frequency, the mean power of the interval from 50 ms before stimulus onset until stimulus presentation was considered as a baseline level. The power change in each frequency band post‐stimulus was expressed as the relative percentage change from the pre‐stimulus baseline.

#### Eyeblink detection

We identified eyeblink responses from the infra‐orbital OPM sensors using the following pipeline: we began with data filtered between 0 and 200 Hz. First, a notch filter at 50 Hz was applied to remove power line noise. Second, a high pass filter at 25 Hz and then a low pass filter at 80 Hz were used. Thereafter, we performed full wave rectification and a final low pass filtering at 20 Hz. A blink peak was identified by the highest amplitude in a trial. For data presentation only, we averaged across 10 consecutive trials.

#### EMG data analysis

EMG Data were high‐passed filtered at 40 Hz and rectified. Data were then epoched and concatenated as had been performed for the OP‐MEG data. Both average waveforms and time–frequency decompositions were examined and compared with OP‐MEG data.

#### Comparing OP‐MEG to eyeblink and EMG data

We examined the trial‐by‐trial temporal correspondence between the activity of OP‐MEG and non‐neural electrical sources, including eyeblinks and muscle potentials, using Pearson's *r*‐values (Yuval‐Greenberg *et al*. 2008). The latency of the maximal power of 5–80 Hz OP‐MEG time–frequency spectrum was computed for each trial and then correlated with peak blink latency. The relationships between OP‐MEG and EMG peak latencies as well as amplitudes were also determined. In addition, EMG and OP‐MEG trials were sorted in order of the latency of peak EMG, to further inspect the connection between these two types of data.

### OP‐MEG source localization

We evaluated the locus of the average evoked response using a dipole fit analysis and the induced power change using a beamformer. In both cases the volume conductor model was the Nolte single shell model (Nolte, 2003), implemented in SPM12, using the scalp boundary from the individual T1‐weighted MRI.

#### Dipole fitting

We reconstructed sources of the evoked field data for each subject using the SPM implementation of equivalent current dipole fitting (Kiebel *et al*. 2008). In brief, the inversion scheme assigned initial means and variances of dipole positions and moments (also called ‘priors’). The final dipole locations and moments were optimized using variational free energy (Friston *et al*. 2007; Penny, 2012; López *et al*. 2014). Free energy quantifies a model's ability to explain the same data (by maximizing the likelihood, similar to other dipole fitting routines) while penalizing excessive optimization (heuristically speaking, a complexity penalty). Moreover, models in which the sources have different prior locations can be compared using free energy. This is particularly useful for comparing different anatomical models of the same data (e.g. is the source more likely to have arisen from the cerebellum or the neck muscles?)

We specified the initial mean locations of six single dipole models based on the literature: (1) right somatosensory cortex (S1), face area, (2) left S1, face area (Nevalainen *et al*. 2006), (3) right cerebellum (in lobule VI), (4) left cerebellum, lobule VI (Cheng *et al*. 2008), (5) right and (6) left medial parieto‐occipital areas. Model 5 and 6 were used because these areas are not only spatially close to the cerebellum but also known to present blink‐related activity (Bardouille *et al*. 2006; Liu *et al*. 2017). We also tested if the evoked responses might have originated from non‐neural sources (i.e. posterior neck muscles) using two additional initial mean locations at the right and left posterior neck. The standard deviation of each prior dipole location was set to 10 mm; the mean and standard deviation of each prior moment were assigned as 0 and 10 nA m, respectively. To avoid local maxima, 50 iterations, with starting locations and orientations (i.e. the means and standard deviations of prior locations and moments) randomly sampled from prior distributions, were carried out for each model in each subject (50 × 6 = 300 iterations in each subject). For each subject, we used the model parameters corresponding to the highest free energy value across all iterations. The maximal free energy values were then averaged across subjects.

#### Beamforming

We used the scalar version of a linear constrained minimum variance beamformer algorithm implemented in the DAISS toolbox for SPM (https://github.com/spm/DAiSS) to localize the source of induced spectral power changes. Two different covariance windows were chosen according to observed sensor level responses (Fig. 4). We first used a covariance window of 5–80 Hz and a time window of −50 to +125 ms and contrasted the 75–125 ms post‐stimulus period with a pre‐stimulus period between −50 and 0 ms. Second, we used a covariance window of 5–30 Hz and a time window spanning −400 to +900 ms and contrasted an individually based 400 ms post‐stimulus peak power period with a pre‐stimulus baseline between −400 and 0 ms. The regularization rate λ was set to be 0 (i.e. unregularized) (Barnes *et al*. 2004). The source orientation was set in the direction of maximal power. The reconstruction grid spacing was 10 mm.

## Results

### Sensor level responses

**Figure 2:**
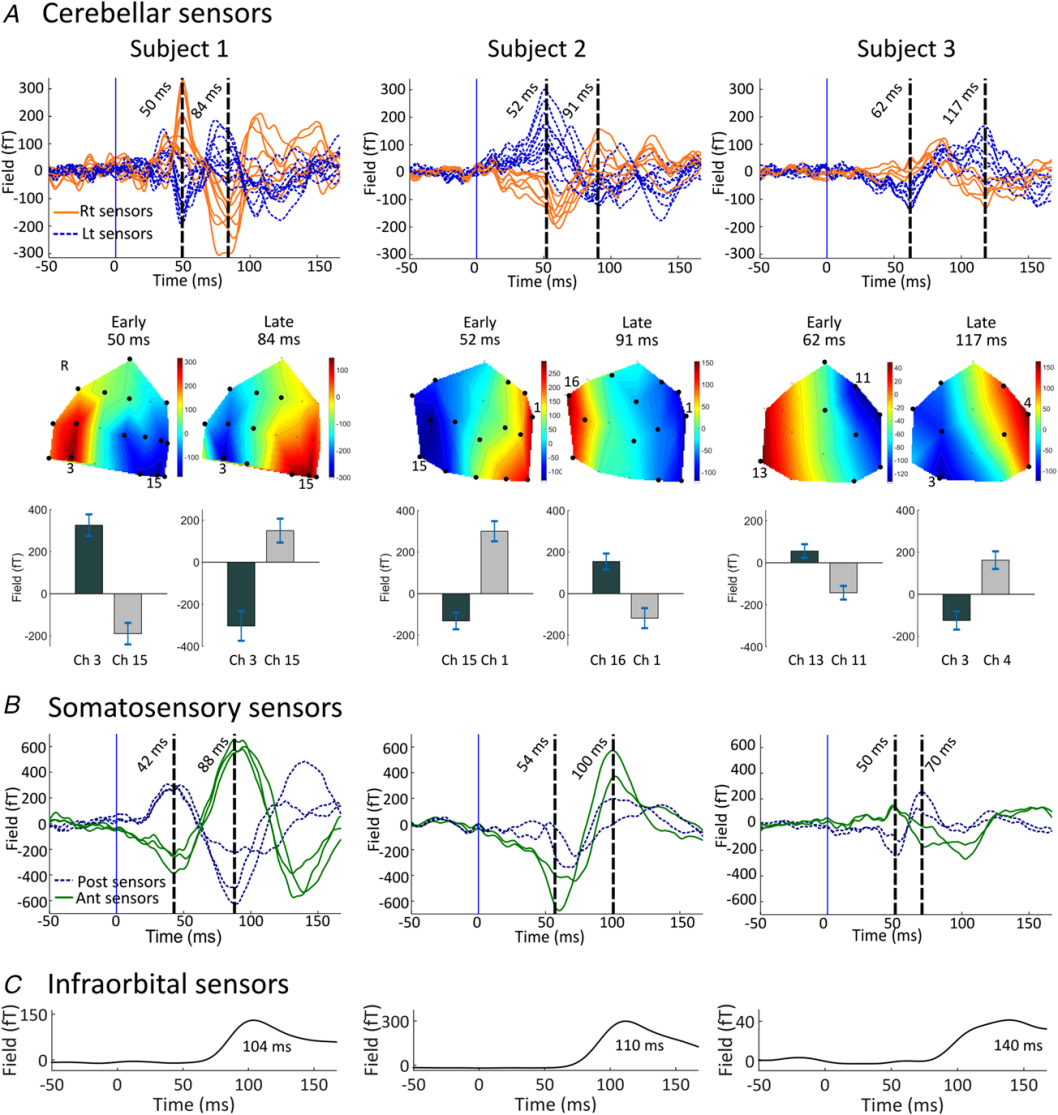
Sensor‐level evoked and blink responses following air‐puff stimulation *A*, upper panel: air‐puff evoked responses over the cerebellar sensor array for 3 subjects. Each trace corresponds to the average signal for one sensor over the posterior cranium, situated left (blue dotted curves) and right (orange continuous curves) of the midline respectively. Middle panel: field maps of the evoked field at the latencies of the two distinct peaks. On the field maps, all sensors at which the response was statistically significant are displayed (black circles). Statistical significance was assessed with paired *t* test comparing mean amplitudes between peak latency ±2 ms and baseline (−4 ms to 0 ms relative to air‐puff contact, *P* < 0.05). The positions of the sensors with the highest activity are highlighted with their channel numbers. Note different sensor layout for each subject. Lower panel: the bar graphs and error bars display the mean field strengths and 95% confidence intervals of the early and late peaks. For each response, the two sensors showing the highest positive and negative activity are displayed. *B*, sensor‐level evoked responses over the right (i.e. contralateral) somatosensory area (anterior and posterior sensors as green continuous and blue dashed curves, respectively). *C*, the average blink waveform obtained from the infra‐orbital sensor, constituting of a single component peaking later than 100 ms. Note the time courses of somatosensory and infraorbital waveforms are distinctly different from those observed at posterior sensors.

We looked at the average evoked response to air‐puff stimulation from the cerebellar OPM sensors and from the sensors positioned over the contralateral somatosensory cortex. Figure 2 *A* shows the average evoked response measured in posterior sensors (left sensors: blue dotted curves, right sensors: orange continuous curves) for the three subjects. Two main peaks were observed across subjects at around 50–60 ms and 85–115 ms. The field patterns observed at these peaks were qualitatively dipolar (Fig. 2 *A* middle panel). For both early and late responses, a paired *t* test showed that peak field strengths were significantly different from those during the baseline time windows in the majority of sensors (for subject 1, 16/19 channels for the early peak, 11/19 for the late peak; for subject 2, 15/18 and 12/18, respectively, and for subject 3, 8/13 for both early and late peaks). There was a slight latency difference (approx. 2 ms on average) between positive and negative going extrema (e.g. subject 2, Fig. 2 *A* upper panel), indicating a more complex source distribution. This small offset was not considered when statistically comparing the peak field strengths. We also observed two response peaks from sensors close to the primary somatosensory cortex (anterior sensors: green continuous curves, posterior: blue dashed curves, Fig. 2 *B*). For each subject, the earliest distinct somatosensory evoked response peaked at 40–50 ms post‐stimulus, compatible with the p45m response to facial tactile stimuli (Nevalainen *et al*. 2006).

**Figure 3:**
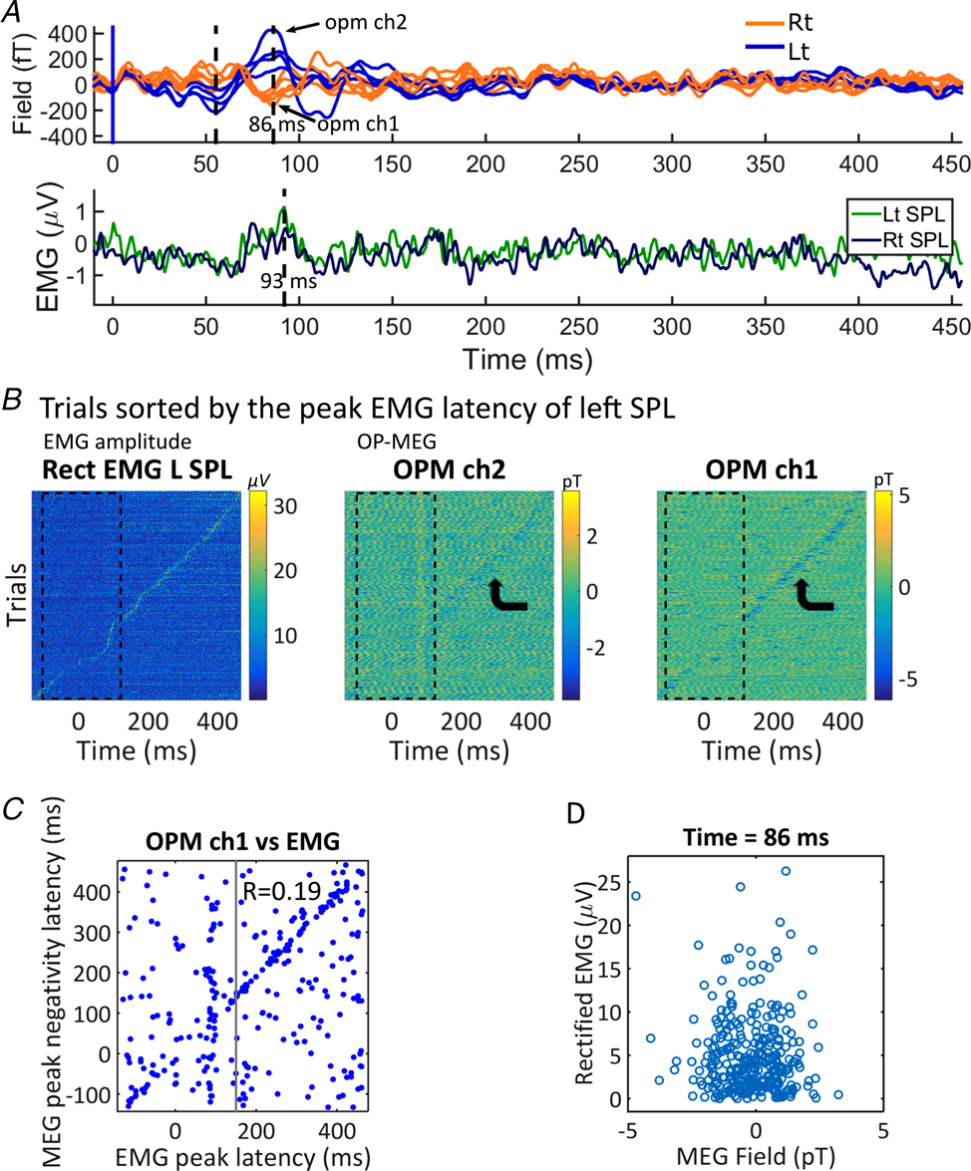
Comparing cerebellar MEG evoked responses with neck EMG data *A*, comparison of the average posterior waveforms (upper) and rectified EMG (lower) of subject 3: on average, EMG activity peaks (93 ms) later than the evoked responses of OP‐MEG, though close to the late component (86 ms). Note that the OP‐MEG evoked responses appear before 150 ms. *B*, EMG and OP‐MEG trials sorted by the latency of peak EMG activation, subject 3. The colour scale represents the amplitude of EMG and OP‐MEG. Peak EMG latencies (left panel) were approximately uniformly distributed across the epoch. Although the MEG negativity peak seems to temporally correlate with the peak EMG in some sensors (6 out of 11, 2 examples shown here), this correspondence is only observable outside the time window of evoked responses (i.e. before air‐puff stimulation and after 150 ms, see bent arrows). *C*, we performed correlation of latencies separately on trials with EMG peaking before and after 150 ms. Pearson's *r* for trials with EMG peaking before 150 ms (i.e. within the time window of evoked responses) is not significant (*r* = 0.12, *P* = 0.16). For trials peaking after 150 ms, there is a weak but significant correlation (*r* = 0.19, *P* = 0.01). *D*, there is no correlation between rectified EMG and OP‐MEG amplitude (OPM ch1) at the time of late MEG peak (*r* = −0.06, *P* = 0.30). [Color figure can be viewed at wileyonlinelibrary.com]

The latency and shape of the blink trace recorded from infra‐orbital sensors were dissimilar to the cerebellar evoked responses (Fig. 2 *C*). Although the late peak of the OP‐MEG evoked responses (Fig. 3 *A* upper, 86 ms) was temporally close to average EMG activity peak (Fig. 3 *A* lower, 93 ms), several other features of cerebellar evoked responses were clearly different from muscle activity. We sorted trials by the latency of peak EMG and showed that peak EMG latencies (Fig. 3 *B*, left panel) were approximately uniformly distributed across the epoch. The EMG peaks seemed to temporally correlate with enhanced negativity of the OP‐MEG, regardless of the polarity of the evoked response, in some sensors (6 out of 11, two examples shown in Fig. 3 *B*, middle and right panels). For example, peak EMG coupled with increased negativity in both OPM channel 1 and 2 (Fig. 3 *B*, middle and right panels), while their evoked responses were opposite in polarity (Fig. 3 *A*, upper panel). Importantly, the EMG–MEG correspondence was only observable outside the time window of evoked responses (i.e. before air‐puff stimulation and after 150 ms). We correlated EMG and OP‐MEG (negative) peak latencies separately for trials with EMG peaking before and after 150 ms. We found only trials peaking after 150 ms had a significant but weak correlation (Fig. 3 *C*; trials with EMG peaking after 150 ms: *r* = 0.19, *P* = 0.01; trials peaking before 150 ms: *r* = 0.12, *P* = 0.16). No correlation between EMG and OP‐MEG amplitude was found at the latency of the late evoked component (*r* = −0.06, *P* = 0.30) (Fig. 3 *D*).

**Figure 4:**
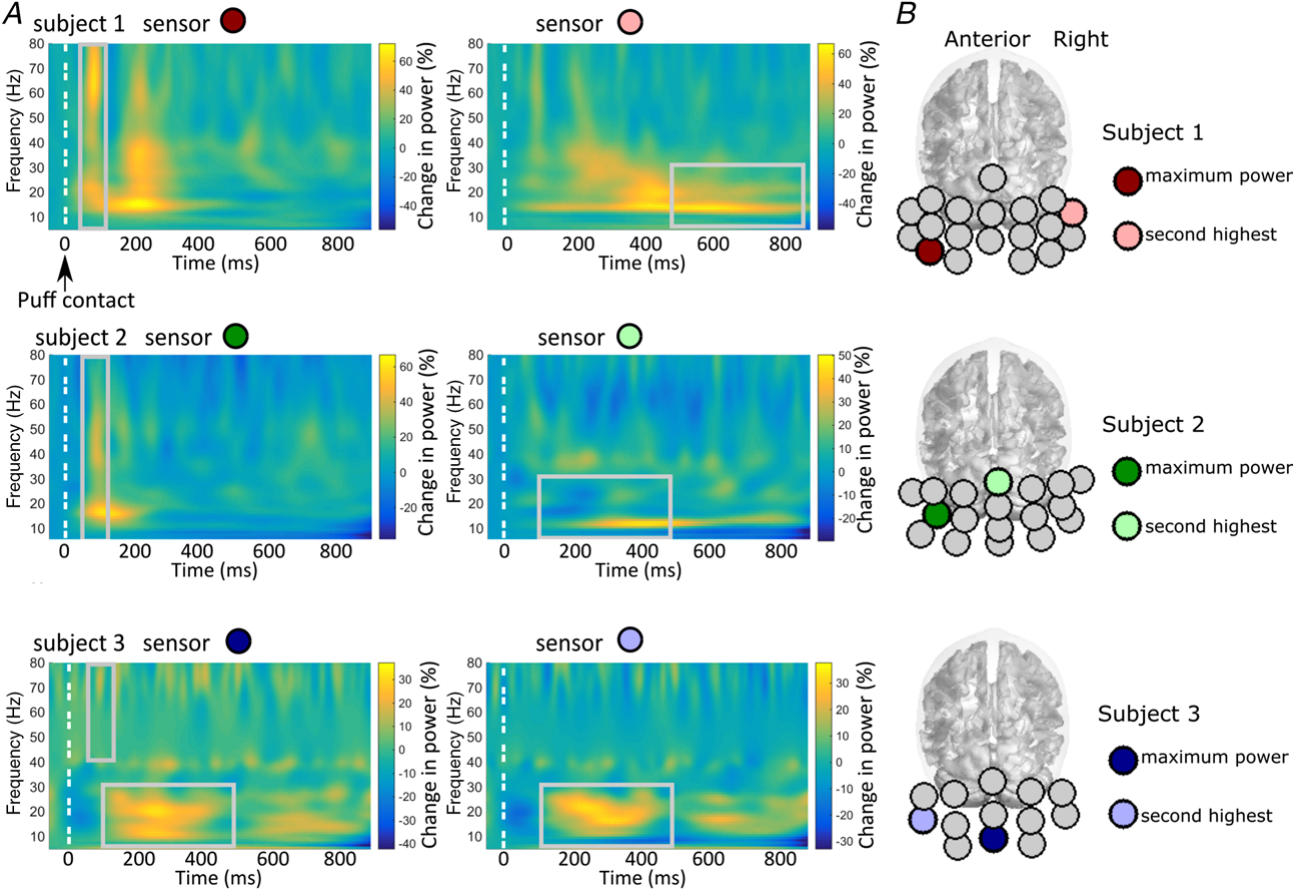
Time–frequency spectrograms showing induced power changes to air‐puff stimulation *A*, each subject's average across all trials at the two sensors with the maximal power changes. *B*, the positions of these sensors marked as coloured circles for each subject and mapped onto the Montreal Neurological Institute (MNI) template brain. Two types of enhanced activity can be observed: (1) a transient broadband response peaking at ∼100 ms (left column) and (2) increased power at 10–30 Hz (alpha and beta range) occurring during a 100–900 ms time window with individual latency differences (right column).

**Figure 5:**
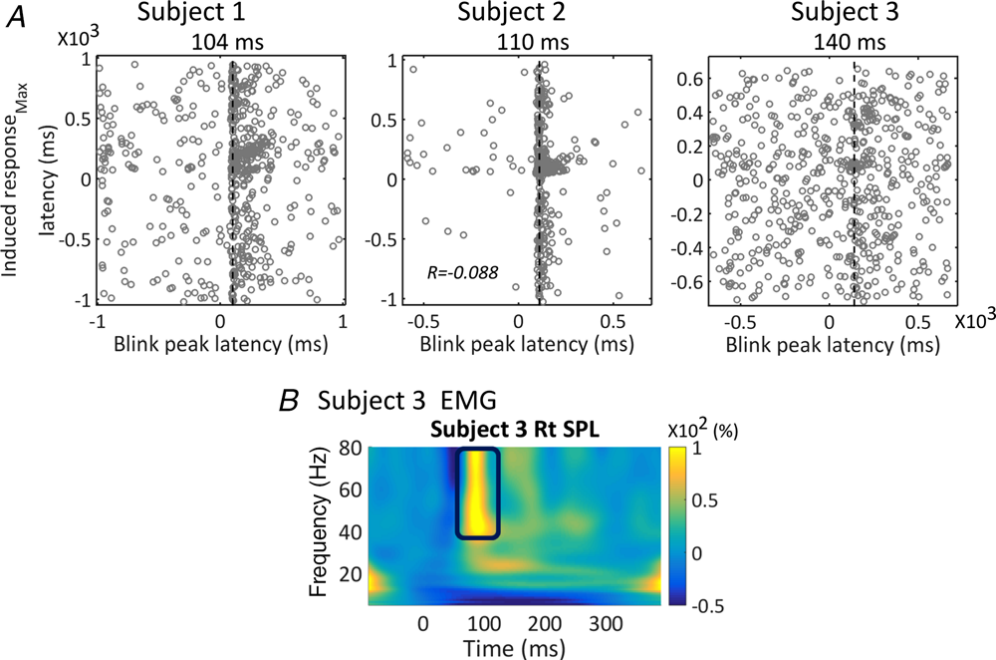
Comparing cerebellar MEG induced responses with blink and neck EMG data *A*, the relationship between latencies of maximal induced response and latencies of blink peaks, as shown by the scatter plot for all trials in each subject. For each trial, the latency of the absolute maximal peak within the 5–80 Hz MEG spectrogram data and between approximately −1000 and +1000 ms was determined and plotted against the latency of the blink maximum. Note that these blink maxima are not necessarily time‐locked to the air‐puff stimulus. Subject 2 showed a significant (*P* = 0.03) but extremely small Pearson *r* value (*r* = −0.088; *r* ^2^ = 0.0078; meaning that less than 1% of the variance is explained by the linear model of blink and MEG peak latency). Correlations were not significant in the other two subjects (subject 1: *r* = 0.007, *P* = 0.87; subject 3: *r* = 0.053, *P* = 0.20). The vertical line of each graph represents the modal latency of the maximum in the blink signal for each subject. Note that they are later than the second MEG component of each individual (as can also be seen in Fig. 2). *B*, time–frequency spectrograms of rectified EMG at the splenius capitis (SPL) of subject 3. A transient broadband increased power at gamma range at ∼100 ms post‐stimulation can be seen. [Color figure can be viewed at wileyonlinelibrary.com]

We also looked for induced spectral changes in the cerebellar sensors. Figure 4 shows the average time–frequency spectrograms (as percentage change of power from baseline) for the two sensors with the largest power change in each subject. Two types of induced responses can be observed. One was a transiently increased broadband power, peaking at ∼100 ms post‐air‐puff contact (Fig. 4 *A*, left column). The other was an enhanced activity at ∼10–30 Hz during 100–900 ms post‐air‐puff contact (Fig. 4 *A*, right column). The correlation across trials between the latencies of the maximal induced power changes and of peak blinks was only significant in subject 2 (*P* = 0.03) and was very weak (*r* = −0.088; *r* ^2^ = 0.0078, which meant that less than 1% of the variance is explained by the linear model of blink and MEG peak latency) (Fig. 5 *A* middle column). However, the time–frequency spectra of EMG show a transient enhanced gamma band power at ∼100 ms (Fig. 5 *B*), which was similar to the transient increased broadband response observed in the OP‐MEG (cf. Fig. 4 *A*, left column).

### Source reconstruction

#### Evoked responses

**Figure 6:**
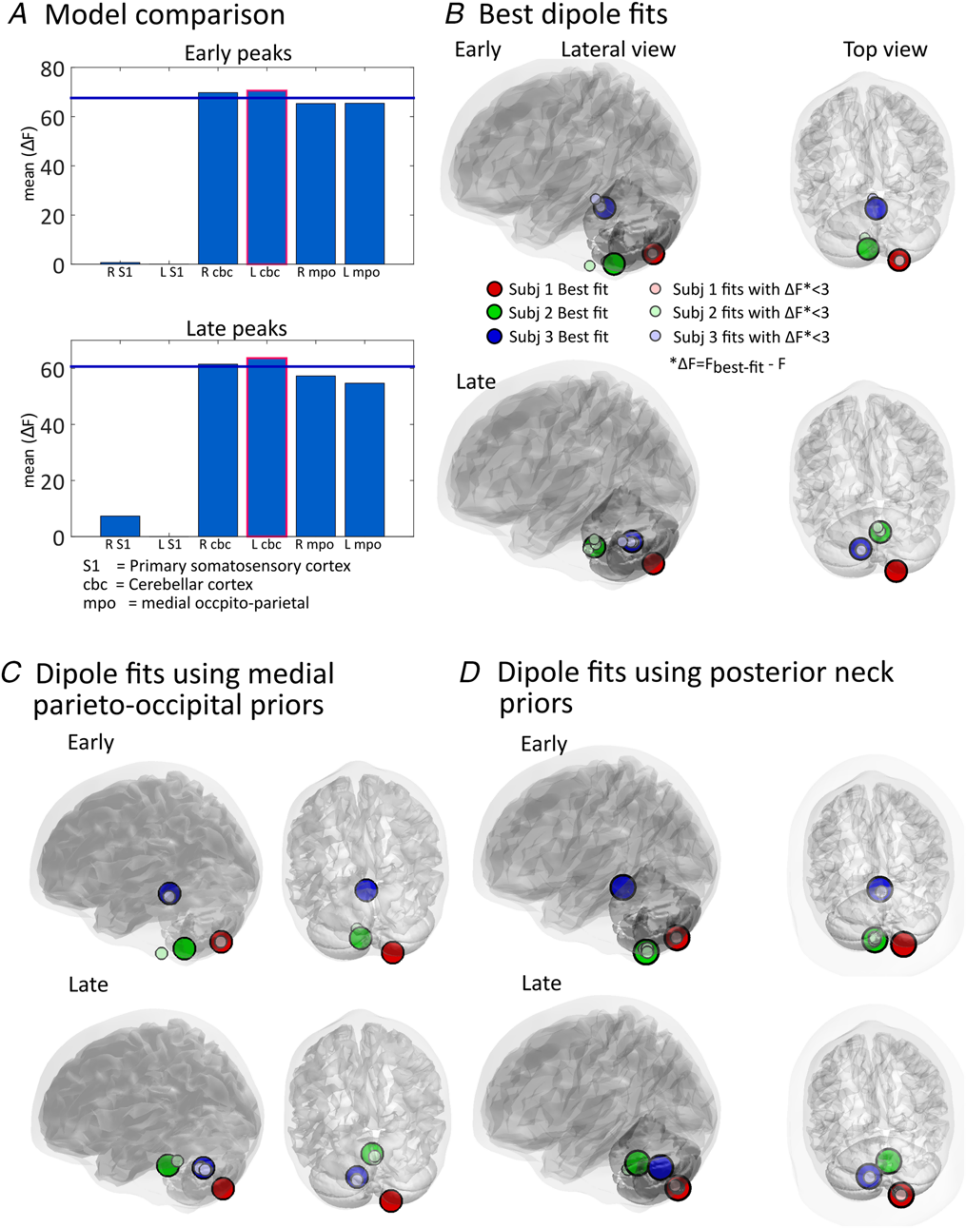
Evoked response source localization: single dipole fits using subject‐specific early and late peaks of evoked responses *A*, model comparison. Free energy (*F*) is used to approximate the model evidence of a given source solution. Bars represent the mean free energy value relative to the poorest model (which was left S1 for both early and late peaks). The left (ipsilateral) cerebellum has the highest model evidence for both early and late peaks. Note that the right cerebellum model is not significantly inferior to the left cerebellum model (bars are not below the Δ*F* = 3 blue line). *B*, single dipole fits for each participant. Large circles represent the source locations of fits with the highest evidence for each individual. Smaller circles are fits which are suboptimal but not significantly so i.e. ∆*F* = (*F*_best‐fit_ − *F*) < 3. *C*, single dipole fits for each peak and subject when using priors in either left or right medial parieto‐occipital area. *D*, single dipole fits for each peak and subject using posterior neck priors. As in *B*, large circles in both *C* and *D* represent the best fits for each subject and small circles represent fits which have Δ*F* = (*F*_best‐fit_ − *F*) < 3. The final estimates of source positions in *C* and *D* are largely in agreement with the dipole fits using more carefully selected physiological priors in *B*.

Figure 6 shows the source reconstruction results of the early and late evoked responses. Figure 6 *A* compares the mean free energy values (averaged across subjects), for six models initiated at different source locations, for the early (peak latency ± 2 ms, respectively yielding subject‐specific windows of 48–52, 50–54 and 60–64 ms; top panel) and late responses (82–86, 90–93, and 115–119 ms; bottom panel). Both cerebellar priors were significantly better than the other models (Δ*F* > 3; as *F* approximates the log model evidence, a positive difference of 3 means the preferable model is about 20 times more likely). The left (ipsilateral) cerebellum lobule VI had the highest model evidence for both peaks; although this model was not significantly better than the model of a source in the right cerebellum (Δ*F* = 0.8 for early and Δ*F* = 2.2 for late peaks). Figure 6 *B* shows the fitted source locations for subject‐specific, early and late time windows. The source localizations were within the cerebellum or the brainstem/cerebellar peduncle adjacent to the cerebellum. Table 1 shows the MNI coordinates and anatomical labels of each fit.

**Table 1:** Location of dipole fits for early and late components of the evoked response in 3 subjects (S1–S3)

		Early							Late					
		MNI coordinates			Dipole moments				MNI coordinates			Dipole moments		
		*X*	*Y*	*Z*	*X*	*Y*	*Z*		*X*	*Y*	*Z*	*X*	*Y*	*Z*
S1	R VIII	17.95	−72.7	−55	2.09	4.51	−30.63	R VIII	12.34	−71.1	−52.1	−3.91	−9.75	−7.43
S2	L IX	−5.76	−48.80	−58.98	11.42	−26.85	2.95	R pons	8.4	−38.07	−43.62	−5.43	23.59	5.53
S3	R pons	2.32	−40.85	−21.7	−15.41	27.88	−25.32	L deep nu	−14	−64.63	−35.2	4.50	−16.05	−2.83

Location labels were based on Diedrichsen *et al*. (2009). Latin numerals denote the assignment to lobules.

Dipole locations converged into the cerebellum even when using priors in the medial parieto‐occipital areas (model 5 and 6, see Fig. 6 *C*). We also undertook additional single dipole fitting using extra‐cranial priors at the posterior neck, approximating the SPL muscle, and found similar results (Fig. 6 *D*).

#### Induced responses

In order to locate the two types of sensor‐level induced power changes, we performed the beamforming analysis with two different covariance windows. For the transient broadband response, a window covering a 5–80 Hz frequency band and a time range of −50 to +125 ms was used, and we contrasted the power between time windows of +75 to +125 ms and −50 to 0 ms. For the response in alpha and beta range, a window covering a 5–30 Hz frequency band and a time range of −400 to +900 ms was used, and we contrasted an individual peak‐based 400 ms poststimulus period (500–900 ms for subject 1, 100–500 ms for subject 2 and 3, see Fig. 4 *A*) with a pre‐stimulus baseline between −400 and 0 ms. For the broadband component, the global maxima were located at the posterior neck. For the induced response in alpha and beta bands, the sources were localized in the medial occipital area. Table 2 shows the MNI coordinates of the global maxima.

**Table 2:** Location of global maxima for broadband and alpha and beta band induced responses in three subjects (S1–S3)

		Broadband MNI coordinates				Alpha and beta MNI coordinates		
		*X*	*Y*	*Z*		*X*	*Y*	*Z*
S1	L neck	−1.25	−68.34	−70.23	R Occipital	0.05	−90.59	−7.60
S2	L neck	−5.81	−41.18	−63.58	R Occipital	29.86	−56.58	6.79
S3	R neck	12.55	−88.93	−63.53	R Occipital	21.71	−68.16	1.61

## Discussion

We have demonstrated that a small OPM array with less than 20 sensors can detect cerebellar evoked responses during unconditioned eyeblinks elicited by brief air‐puffs. The evoked responses had early (∼50 ms) and late components (∼90–110 ms). The source localization of these evoked responses was in the ipsilateral cerebellum, generally consistent with previous neural recordings in animals and fMRI in humans.

MEG sensors can most readily detect signals when neuron populations aligned in parallel and oriented tangentially to the overlying skull fire synchronously (i.e. ‘open‐field neurons’). The mammalian cerebellum is characterized by extensive and fine‐scale foliation and has a patchy, fractured spatial organization of inputs (Tesche & Karhu, 1997). So it is likely a proportion of concurrent electric currents will cancel each other out due to opposite orientations (i.e. ‘close‐field neurons’), similar to what has been observed in the visual cortex (Vanegas *et al*. 2013). As a result, cerebellar MEG signals are expected to be considerably smaller, if not undetectable, compared to neocortical signals. In fact, this was one of the main reasons that cerebellar activity used to be considered unobtainable using MEG. However, realistic simulations and experimental data support that, though challenging, sources with this kind of close‐field neuronal organization can be identified in MEG (Hashimoto *et al*. 2003; Attal & Schwartz, 2013; Krishnaswamy *et al*. 2017). Presumably, by minimizing sensor‐to‐brain distances and co‐registration errors, OP‐MEG technology should enhance the detectability of even these weak signals, and our results provided empirical evidence supporting this assumption.

In the small number of published MEG studies, cerebellar activation to somatosensory stimuli has been identified by either using *a priori* assumptions of an equivalent current dipole in the cerebellum (Tesche & Karhu, 1997, 2000) or by using a beamforming source reconstruction (Hashimoto *et al*. 2003). Hashimoto *et al*. (2003) showed a four‐component response in the cerebellum following electrical stimulation of the median nerve, and the authors made a putative assignment of these peaks to different cerebellar inputs. The Hashimoto study showed robust source‐level images, predominantly localizing to ipsilateral medial cerebellum, but was based on 10,000 trials. By contrast, our results are apparent at sensor level and are based on 550–580 trials per participant. We expect that a further reduction in required trials could be achieved by the bespoke design of a cerebellar helmet, to both increase sensor numbers and optimize their locations to further increase signal‐to‐noise.

Due to the nature of the eyeblink paradigm, there were eye and eyelid movements in almost every trial. We thus examined in detail the relationship between infra‐orbital blink signals and OP‐MEG data to understand if ocular sources might contribute to the MEG sensor‐level activity (even though artefacts from the corneo‐retinal dipole and extra‐orbital muscles have been shown to be highly focal and limited to fronto‐central sensors in the MEG; Carl *et al*. 2012; Muthukumaraswamy, 2013). We found that eyeblink occurred relatively late (∼100–140 ms) after stimulus onset (Fig. 2 *C*) and, importantly, peaked after the later cerebellar component for each subject. Blink is therefore an improbable source of these evoked responses. On a trial‐by‐trial basis, we found no or only a very weak temporal correlation between the latencies of the maximal spectral power changes and blinks (Fig. 5 *A*). Thus, the induced responses were also not likely to be products of blinks.

Another source of non‐neural artefact to be considered is neck muscle, because it is near the cerebellum and a sudden air‐puff does lead to some reflex head motion, visible in early acclimatization trials. However, the evoked responses are unlikely to be caused by muscle activity, as dipole fits converged to the cerebellum even when using posterior neck priors (Fig. 6 *D*). A weak temporal correlation (Pearson *r* = 0.19, *P* = 0.01) between EMG and OP‐MEG activity broke down during the time window of evoked responses (*r* = 0.12*, P* = 0.16; Fig. 3 *C*). Further, there was no correlation between the amplitude of the peak surface EMG and the OP‐MEG response (*r* = −0.06, *P* = 0.30; Fig. 3 *D*). Regarding the induced responses, the time–frequency spectra of neck muscle EMG (Fig. 5 *B*) revealed a transient broadband component at ∼100 ms and 40–80 Hz, whose time and frequency features resemble the broadband induced response in the OP‐MEG. Beamforming analysis also localized the source of this transient enhancement of gamma band at the posterior neck. We thus conclude that this broadband induced response was likely to be due to muscle artefacts.

To our knowledge, this is the first MEG study examining the neural response due to the unconditioned stimulus (US, i.e. air‐puffs) in an eyeblink conditioning paradigm. Being a model system for cerebellar learning, the neural circuits of both unconditioned and conditioned blink responses have been extensively investigated in animals. In untrained rodents, single‐unit recordings from Purkinje cells in ipsilateral cerebellar cortical lobule VI show that air‐puffs elicit climbing fibre inputs, with high probability, peaking around 15–50 ms post‐stimulus (Mostofi *et al*. 2010; Ohmae & Medina, 2015). Importantly, the excitation of the very powerful climbing fibre–Purkinje cell synapse, simultaneously activating the dendrites of a spatially aligned set of Purkinje cells, has been considered to be the most probable source for a strong open field detected by MEG recordings (Tesche & Karhu, 1997). According to local field potential recordings in animal models, the US reaches the cerebellum first via an early mossy fibre response and then the aforementioned climbing fibre response (Hesslow, 1994; Mostofi *et al*. 2010). Our OP‐MEG evoked responses showed two peaks, the first tightly clustered around 50–60 ms while the second occurred 80–110 ms post‐puff with significant inter‐individual differences in latency. These first human data are thus broadly consistent with the animal literature, and the climbing fibre signal might contribute to the early 50 ms response. Previously, similar qualitative features have been seen in human MEG responses to simple somatosensory input, including multiple components (Tesche & Karhu, 2000; Hashimoto *et al*. 2003) and inter‐individual latency variability (Tesche & Karhu, 1997). Hashimoto *et al*. speculated that a component at 50–70 ms was driven by the median‐nerve‐induced activation of climbing fibres.

Although the inversion scheme suggested the left (ipsilateral) lobule VI was the best fitting single‐dipole model of both evoked responses, it should be noted that the best fits located close to the midline (see Table 1), more medial in all three subjects than the lobule VI, the location expected from the literature (Dimitrova *et al*. 2002; Cheng *et al*. 2008; Thurling *et al*. 2015). Besides, for both the early and late peaks of the evoked field, the differences in free energy between single dipoles in the right and left cerebellum lobule VI were small (∆*F* < 3, Fig. 6 *A*). One potential cause of this finding is that although the ipsilateral lobule VI is the dominant neuronal source of the US signal, it is not the only cerebellar focus being activated by it. Concurrent (but weaker) neuronal activation to US stimuli has been found in the contralateral lobule VI, bilateral lobule VIIb/Crus I/VIIIa (the posterior region of the hemisphere) as well as bilateral deep nuclei (Dimitrova *et al*. 2002; Mostofi *et al*. 2010; Thurling *et al*. 2015), so that a two‐ or multiple‐dipole model might be more accurate. When fitting the evoked fields with two‐dipole priors, the majority of best fits were indeed paired bilateral cerebellar sources (figure not shown here). These results support the hypothesis of bilateral, and maybe multi‐focus, activation in the cerebellum by unilateral air‐puff stimulation. This said, we admit the current results cannot conclusively locate the sources at a resolution of individual cerebellar lobules (Schmahmann *et al*. 1999).

In this proof‐of‐principle study, we also searched for induced responses to air‐puff stimulation. At the sensor level, two types of induced responses were observed: a brief increased broadband power at ∼100 ms and an increased power in a lower‐frequency range (10–30 Hz) between approximately 100 and 900 ms. However, as has been mentioned, the source of the former component was found to be outside the skull and likely to be caused by neck muscle activity. While we could not observe any overt head motion, the head was freely supported, with neck muscles activated and it is possible that reflex responses to the air‐puff stimulus caused this effect. On the other hand, the alpha and beta band responses were located in the medial occipital area. It is possible that these lower‐frequency components reflect neural responses to the transient changes of visual input during blinks, as has been reported previously (Bardouille *et al*. 2006; Liu *et al*. 2017). However, further studies will be needed to confirm the nature of this induced lower band response.

The number of OPM sensors available has limited our current tests with dipole localization accuracy (and detectability) decreasing sharply in regions outside the sensor coverage (Mosher *et al*. 1993). Source localization would thus likely benefit from a denser and spatially extended sensor coverage, especially given the relatively greater depth of the cerebellum, its complex architecture, and the diffuse spatial signature seen in the evoked responses (see the field maps in Fig. 2 *A*, middle panel). With the next generation of smaller and lighter OPM sensors, we expect to design whole‐head helmets/caps to address this issue. Additionally, it would be useful to design helmets that allow sensors to be placed over the upper neck to provide greater coverage of the inferior cerebellum, which was just below the lower margin covered by the current set‐up. We are also planning to perform identical cerebellar measurements using both whole‐head OP‐MEG and SQUID‐MEG systems, which will provide a fair comparison regarding signal magnitudes and source localization. At the moment we believe such a direct comparison would unfairly prejudice the relatively low spatial resolution available to us, with less than 20 OPM sensors. Finally, to give confidence in the identities and localizations of our findings, we intend to extend our studies to include classical conditioning and extinction of the eyeblink and thus aim to track the predicted changes in the responses to the unconditioned and conditioned stimuli across the learning and extinction phases (Ohmae & Medina, 2015).

### Conclusion

We have demonstrated an OP‐MEG system that can be used to study the electrophysiology of the human cerebellum. The similarities between human MEG and animal field potential data in this proof‐of‐principle task offer promise for future studies to advance our understanding of cerebellar function through non‐invasive electrophysiology in humans. Possessing sufficient signal detectability and being wearable, and potentially moveable, we expect the OP‐MEG system to be a powerful tool in the investigation of cerebellar functions in both motor and cognitive tasks (Sokolov *et al*. 2017; Boto *et al*. 2018; Tierney *et al*. 2018). This OP‐MEG system also has the capacity to fill the ‘white regions’ (Niedermeyer, 2004) of the map of clinical electrophysiology in the cerebellum, with impacts on diseases including movement disorders (Bostan & Strick, 2018), mental disorders (Romer *et al*. 2018) and dementia (Fyfe, 2016), to name a few.

## Additional information

### Competing interests

This work was funded by a Wellcome award which involves a collaboration agreement with QuSpin. QuSpin built the sensors used here and advised on the system design and operation, but played no part in the subsequent measurements or data analysis.

### Author contributions

C.L., G.R.B. and R.C.M. contributed to the conceptual design of the experimental work, acquisition and interpretation of the data, and drafting/revising the manuscript. T.M.T., N.H. and E.B. contributed to the acquisition and interpretation of the data and assisted with the manuscript revisions. J.L., S.B., R.B. and M.J.B. contributed to the analysis, interpretation of the data and assisted with the manuscript revisions. All authors have read and approved the final version of this manuscript and agree to be accountable for all aspects of the work in ensuring that questions related to the accuracy or integrity of any part of the work are appropriately investigated and resolved. All persons designated as authors qualify for authorship, and all those who qualify for authorship are listed.

### Funding

C.L. was funded by a BBSRC research grant (BB/M009645/1) and by Wellcome grant WT212422. This work was also supported by a Wellcome collaborative award to G.R.B., M.J.B. and R.B. (203257/Z/16/Z, 203257/B/16/Z). The WCHN is supported by a strategic award from Wellcome (091593/Z/10/Z). R.C.M. was funded by a Royal Society Leverhulme Senior Fellowship and by Wellcome grant WT212422.
